# Serotherapy as Graft-Versus-Host Disease Prophylaxis in Haematopoietic Stem Cell Transplantation for Acute Lymphoblastic Leukaemia

**DOI:** 10.3389/fped.2021.805189

**Published:** 2022-01-06

**Authors:** Steven J. Keogh, Jean-Hugues Dalle, Rick Admiraal, Michael A. Pulsipher

**Affiliations:** ^1^Cancer Centre for Children, The Children's Hospital at Westmead, Westmead, NSW, Australia; ^2^Hôpital Robert Debré, GHU AP-HP. Nord Université de Paris, Paris, France; ^3^Princess Maxima Center for Pediatric Oncology, University Medical Centre Utrecht, Utrecht, Netherlands; ^4^Children's Hospital Los Angeles, Cancer and Blood Disease Institute, Los Angeles, CA, United States

**Keywords:** serotherapy, anti-T-lymphocyte globulin, anti-thymocyte globulin, alemtuzumab, acute lymphoblastic leukaemia, pharmacokinetics, graft-versus-host disease (GVHD), haematopoietic stem cell transplant

## Abstract

Serotherapy comprising agents such as anti-thymocyte globulin, anti-T-lymphocyte globulin, and the anti-CD52 monoclonal antibody alemtuzumab is used widely to reduce the incidence of graft-versus-host disease (GvHD) after paediatric haematopoietic stem cell transplantation (HSCT). The outcome of transplants using matched unrelated donors now approaches that of matched sibling donors. This is likely due to better disease control in recipients, the use of donors more closely human-leukocyte antigen (HLA)-matched to recipients, and more effective graft-versus-host disease (GvHD) prophylaxis. The price paid for reduced GvHD is slower immune reconstitution of T cells and thus more infections. This has led to studies looking to optimise the amount of serotherapy used. The balance between prevention of GvHD on one side and prevention of infections and relapse on the other side is quite delicate. Serotherapy is given with chemotherapy-/radiotherapy-based conditioning prior to HSCT. Due to their long half-lives, agents used for serotherapy may be detectable in patients well after graft infusion. This exposes the graft-infused T cells to a lympholytic effect, impacting T-cell recovery. As such, excessive serotherapy dosing may lead to no GvHD but a higher incidence of infections and relapse of leukaemia, while under-dosing may result in a higher chance of serious GvHD as immunity recovers more quickly. Individualised dosing is being developed through studies including retrospective analyses of serotherapy exposure, population pharmacokinetic modelling, therapeutic drug monitoring in certain centres, and the development of dosing models reliant on factors including the patient's peripheral blood lymphocyte count. Early results of “optimal” dosing strategies for serotherapy and conditioning chemotherapy show promise of improved overall survival.

## Introduction

In allogeneic haematopoietic stem cell transplantation (HSCT) for acute lymphoblastic leukaemia (ALL) the term serotherapy is used to describe inclusion of anti-thymocyte globulin (ATG), anti-T-lymphocyte globulin (ATLG), or alemtuzumab in the conditioning regimen.

Anti-thymocyte serum was developed by HSCT pioneers early in the history of transplantation (1, 2). After being used for the treatment of graft-versus-host disease (GvHD) during the 1970s (3), serotherapy was introduced to some conditioning regimens before allogeneic HSCT in order to induce T-cell depletion in the recipient. This led to enhanced engraftment as well as GvHD prophylaxis by depleting both recipient antigen-presenting cells and donor T cells from the graft (4). The result of ATG administration varies widely depending on product used, dosage, timing (around day-10 vs. just prior to graft infusion), leukocyte count at time of administration and patient age.

Horse ATG, while routinely used in the treatment of severe aplastic anaemia, is currently rarely used in the modern HSCT setting, thus we will not discuss this product here. Two different rabbit ATG products are indicated for GvHD prophylaxis, namely Thymoglobulin^®^ (Sanofi Genzyme, France; G-ATG) and Grafalon^®^ (Neovii, Switzerland, formerly Fresenius ATG or F-ATG). Grafalon^®^ is also referred to as ATLG.

The two rabbit ATG preparations differ in their derivation and, therefore, their effects are different. Thymoglobulin^®^ is obtained from rabbits after administration of human thymocytes, while Grafalon^®^ is obtained from rabbits after administration of a specific immortalised T-cell line: Jurkat cells (5). Both products contain polyclonal antibodies directed against many antigens involved in immune cell trafficking and adhesion as well as antigens on T cells, B cells, natural killer (NK) cells, and other immune cells (6). The variety of antibodies and the titres differ between the two products, leading to different pharmacokinetic (PK) and pharmacodynamic (PD) profiles. These differences must be taken in consideration when analysing study data and interpreting study reports. Dosage and scheduling matter since they lead to different serum levels and exposure duration post-HSCT as measured by the area under the curve and other variables.

By contrast, alemtuzumab is a monoclonal antibody that specifically targets CD52 glycoprotein, an antigen expressed on the surface of normal B and T lymphocytes, NK cells, monocytes, macrophages, and some dendritic cells. It was originally developed in the United Kingdom from rat immunoglobulin (Campath-1G) and later was modified to be the first humanised monoclonal antibody, Campath-1H. Originally the formulation was marketed as Mabcampath^®^ and was used to treat CD52^+^ T- and B-cell cancers, notably chronic lymphocytic leukaemias and other lymphocyte-mediated conditions (7). It is currently licenced and formulated for the treatment of relapsing multiple sclerosis as Lemtrada^®^ but is still available for use alongside HSCT in some jurisdictions. As with ATG products, its effects on both donor and recipient immune cells differs depending on dose and timing of administration. Its use is rare outside of the United Kingdom in the context of HSCT for paediatric ALL.

For a review of the approach to GvHD prophylaxis beyond serotherapy as well as the management of patients developing GvHD, including those with steroid-refractory GvHD, see the review by Diaz de Heredia Rubio et al. in the current supplement.

## Practise Differences in the Inclusion of ATG in Conditioning Regimens

One challenge in assessing the role of serotherapy in treating ALL patients has been practise differences, especially notable in North American vs. European trials. Accepted practise in North America, as shown by large, randomised trials, generally omits serotherapy for matched unrelated donors (MUDs). Large randomised trials of the Children's Oncology Group (ASCT0431: a randomised trial of tacrolimus and methotrexate vs. sirolimus, tacrolimus, and methotrexate in patients with ALL in first or second complete remission) and the Bone Marrow Transplant Clinical Trials Network [CTN0201: bone marrow vs. peripheral blood stem cells (PBSCs) in MUD HSCT; CTN0501: 1 vs. 2 cord blood units for ALL or acute myeloid leukaemia (AML)] all omitted serotherapy (8–10). In contrast, large, randomised trials in Europe conducted in a similar time period [ALL-SCT-Berlin-Frankfurt-Münster (BFM)-2003 trial: MUD vs. sibling donors in ALL; For Omitting Radiation Under Majority age (FORUM) trial: total body irradiation (TBI) vs. non-TBI conditioning for ALL] included serotherapy for all MUD recipients (11, 12). While more recent data from serotherapy trials have led to an increase in the use of serotherapy for mismatched unrelated donors (MMUDs) in North America, debate remains about the role of serotherapy in recipients of bone marrow HSCT from fully matched unrelated donors.

## Use of Low-Dose vs. High-Dose ATG

In 2017, Locatelli et al. published the results of a prospective, multicentre, randomised, open-label Phase III trial looking at the efficacy of two different doses of rabbit ATG to prevent GvHD in children with haematological malignancies (aged 0–18 years) undergoing HSCT from MUDs (13). Prior to this study, none of the published trials had focused on a paediatric population. The study was performed in seven Italian centres in collaboration with the HSCT Working Group of the Italian Association for Paediatric Haematology and Oncology (AIEOP). The study used bone marrow and PBSC transplants from unrelated donors matched at ≥6 out of 8 loci. Randomisation was between a total dose of 15 mg/kg and 30 mg/kg of rabbit ATG (Grafalon^®^) given between day-4 and day-2 with myeloablative conditioning. All patients also received GvHD prophylaxis with cyclosporine A. The study found that, in children with haematological malignancies, the use of 15 mg/kg rabbit ATG resulted in better overall survival [OS; 78 vs. 62%, respectively, hazard ratio (HR) 1.80, *p* = 0.045] and event-free survival (77 vs. 61%, respectively, HR 1.87, *p* = 0.028) than that observed with a 30 mg/kg dose. ATG at a dose of 15 mg/kg can spare life-threatening viral infections caused by delayed T-cell reconstitution without significantly increasing the incidence of acute and chronic GvHD and without adversely affecting other outcomes such as engraftment or relapse.

In 2020, Kang et al. published results of a retrospective study also looking at the optimal dosing of ATG for transplantation of children with leukaemia receiving a PBSC graft from a MUD or haploidentical family donor (HFD) (14). The primary aim was to look at OS and relapse rates with secondary aims including evaluation of the severity of acute GvHD, chronic GvHD, and infectious complications that arose because of delayed immune reconstitution. The retrospective cohort of patients was identified from a prospectively enrolled HSCT registry in Seoul, South Korea, between April 2009 and September 2018. Patients underwent first HSCT for leukaemia from MUD or HFD with unmanipulated PBSCs after receiving Thymoglobulin^®^ in the conditioning regimen from day-4 to day-1. From 2009 to 2014, recipients of a MUD graft received 7.5 mg/kg ATG and recipients of an HFD graft received 10 mg/kg ATG. From 2014 to 2018, recipients of an MUD graft received 3.75 mg/kg ATG and recipients of an HFD graft received 5 mg/kg ATG.

Patients with ALL made up 50% of the 78 patients in the low-dose group (3.75–5 mg/kg) and 44.1% of the 118 patients in the high-dose group (7.5–10 mg/kg). Multivariate analysis showed that both the European Society of Bone and Marrow Transplantation (EBMT) disease stage at transplant and ATG dose group (low or high dose) had a significant influence on OS and relapse incidence. The high-dose ATG group had an increased risk of death [HR 2.02, 95% confidence interval (CI) 1.05–3.88, *p* = 0.036] and relapse (HR 1.81, 95% CI 1.03–3.17, *p* = 0.038) compared with the low-dose ATG group. There was no significant difference in the cumulative incidence of acute GvHD or chronic GvHD between the high- and low-dose ATG groups. The high-dose ATG group also had a higher incidence of cytomegalovirus viraemia (70.3 vs. 51.3%, respectively, *p* = 0.007) and Epstein-Barr virus reactivation (81.4 vs. 39.7%, respectively, *p* = 0.001) than the low-dose ATG group.

## Consensus Recommendations on Serotherapy in HSCT

An international expert panel published consensus recommendations on the use of rabbit ATG (i.e., Thymoglobulin^®^ or Grafalon^®^) in HSCT in 2020 (15). They developed the recommendations using the Delphi method with focused review of the role of rabbit ATG based upon published randomised trials, multiple meta-analyses, and expert consensus. The review included both paediatric and adult studies and concluded: (1) Rabbit ATG is indicated for MUD or MMUD bone marrow or PBSC grafts to prevent severe acute and chronic GvHD; (2) rabbit ATG could possibly be of use for related donors, although the data were from a single trial using PBSC; (3) Use of rabbit ATG in reduced-intensity conditioning (RIC) may be appropriate but comes at a cost of an increased risk of relapse; and (4) use of rabbit ATG in haploidentical bone marrow transplantation is regimen specific and the role with cord blood grafts is inconclusive. Specifics about dosing, side effects and post-HSCT management were also reviewed.

A major challenge with developing consensus recommendations relevant to paediatric patients with ALL is that available data are largely drawn from adult studies which used PBSC grafts and, furthermore, studies using cord blood grafts included the use of double cords, which is common in adults and associated with higher rates of GvHD compared with grafts from single cords. Of note, the dosing and PK of rabbit ATG in children can vary significantly from adults (16). These variations lead to different practise in paediatrics vs. adults for myeloablative first HSCT for ALL, where rabbit ATG is generally not used because MSD HSCT is performed using bone marrow (9, 11). For umbilical cord grafts, randomised trials have shown significant increases in acute and chronic GvHD when multiple cord blood units are used (10, 17); however, for single-unit grafts (which are most often used in paediatrics), acceptable rates of acute GvHD and very low rates of chronic GvHD occurred (18). This has led to some experts concluding that serotherapy may not be needed for HSCT when a single cord unit is used. This theory is supported by a meta-analysis (19). However, other experts have argued that, with appropriate timing and dosing of rabbit ATG, this serotherapy could possibly lead to a benefit in umbilical cord HSCT for ALL in children (20).

## Population Pharmacokinetics and Pharmacodynamics of ATG

Dosing of ATG in children was traditionally based on extrapolations of adult dosing. A fixed dose per kilogramme is usually given to children 0–18 years of age. Here, the assumption is made that both the PK and PD of ATG show a linear increase with body weight. An understanding that this is usually not the case led to the investigation of the PK and PD of ATG in children. Since the brands of ATG are not biosimilar, their PK and PD will also not be fully comparable. As such, we will present data below according to each specific brand of ATG.

## Thymoglobulin^®^ (Sanofi Genzyme)

Some studies have investigated ATG concentration–time curves and how these are impacted by anti-ATG antibodies (21). Partly based on their findings, a formal population PK study was performed in children receiving Thymoglobulin^®^ (22). This showed that both body weight and absolute lymphocyte count just prior to the first dose of Thymoglobulin^®^ were good predictors of PK. Absolute lymphocyte counts were included because they are the target for Thymoglobulin^®^ binding and thus affect its clearance.

The overall goal of describing population PK was to develop an individualised dosing regimen for Thymoglobulin^®^ to be used in future patients. With the PK elucidated, the next step was to identify the optimal exposure of Thymoglobulin^®^. The exposure to Thymoglobulin^®^ before graft infusion was found to drive the effects of this serotherapy, while over-exposure after infusion of the graft led to toxicity (14, 23). Higher exposure of Thymoglobulin^®^ after graft infusion was highly correlated with poor or absent early T-cell recovery, which in turn was a strong predictor for treatment-related mortality and viral reactivations. Thymoglobulin^®^ exposure before graft infusion, however, decreased GvHD and graft failure. Moreover, the PD was found to be dependent on stem cell source, as the earlier Thymoglobulin^®^ is administered in conditioning the less antibody is present at the time of HSCT infusion. For example, some approaches have started Thymoglobulin^®^ administration earlier in conditioning for cord blood transplantations.

Based on the population PK model and the determination of the therapeutic window for Thymoglobulin^®^, an individualised dosing regimen has been designed for its use in children (24). The optimal dose for each patient is calculated based on three factors: (1) body weight, (2) baseline lymphocyte counts, and (3) stem cell source. Patients with higher body weights, lower lymphocyte counts, and receiving a cord blood graft are proposed to receive a lower dose in mg/kg compared to patients with lower body weights, higher lymphocyte counts and receiving a bone marrow or PBSC graft. In addition, the first infusion of Thymoglobulin^®^ is given more distally to the HSCT in order to increase the exposure before graft infusion and decrease the exposure after graft infusion. The first dose of Thymoglobulin^®^ is given on day-9 before HSCT for MUD and cord blood grafts.

The efficacy of this individualised dosing regimen has been assessed in a prospective, open-label, Phase II clinical trial. Patients receiving individualised dosing of Thymoglobulin^®^ showed superior T-cell recovery compared with patients receiving fixed dosing, with 83 vs. 54% achieving a CD4 count of >50/mm^3^ at two consecutive timepoints within 100 days after HSCT, respectively. Despite the relatively small sample size, a trend towards improved OS was observed with individualised dosing vs. fixed dosing, and rates of GvHD were the same between groups (24).

## Grafalon^®^ (Neovii)

The current literature on Grafalon^®^ PK and PD remains limited to studies investigating concentration–time curves (23). Some inferences regarding the clearance of Grafalon^®^ and Thymoglobulin^®^ may be imprecise as they are made based on non-compartmental analysis, which in light of the highly non-linear PK properties of ATG may be inaccurate. No population PK models of Grafalon^®^ are available, nor are reports of investigations into the exposure–effect relationship or individualised dosing.

## Therapeutic Drug Monitoring of ATG

While individualised dosing of ATG is more accurate in attaining the desired exposures, there is still some unpredictable variability between patients. To minimise variability, therapeutic drug monitoring (TDM) can be used. An important requirement for quality TDM is a reliable, reproducible, and fast assay for the active fraction of ATG. This may well be the major limiting step for implementing TDM for ATG in most centres. To our best knowledge, the only centre that performs TDM for ATG are several Centres in The Netherlands.

Given that TDM is a costly and time-consuming procedure, at the Princess Maxima Centre, criteria have been developed such that TDM is only performed where there is a high risk for graft failure, GvHD or ongoing infections and in patients who receive a cord blood transplant and need swift immune recovery. Furthermore, patients receiving a second course of ATG could possibly be eligible for TDM to screen for anti-ATG antibodies. As most patients with ALL do not meet these criteria, few have been subject to ATG TDM in clinical practise.

## Pharmacogenetics of ATG

Currently, the role of pharmacogenetics in ATG pharmacology is limited. As no hepatic metabolism is involved in ATG clearance, there is no role of pharmacogenetic variants of the cytochrome p450 or glucuronosyltransferase families. Hypothetically, there may be a role of variants of Fc receptors (FcR), receptors which are part of the recycling process of immunoglobulins including ATG (25). Whether these variants also impact rabbit immunoglobulin G and cause differences in PK between patients remains to be investigated.

## Population Pharmacokinetics of Alemtuzumab and Comparison With ATG

Some studies have reported on the population PK of alemtuzumab. Three of these investigated alemtuzumab in the setting of allogeneic HSCT; one is published only as a conference proceeding (26). In the two published studies, one investigated alemtuzumab PK after relatively high intravenous dosing (27), and one investigates both the PK and PD of alemtuzumab following subcutaneous dosing (28). Both identify only body weight as a predictor of alemtuzumab PK, not lymphocyte counts as was previously found in ATG PK (22). Explanations may be that most children in the intravenous study received a relatively high dose of alemtuzumab, thereby introducing an excess of drug in relation to target. The authors of the paper on subcutaneous alemtuzumab (28) suggest the limited number of patients in their studies caused the lack of relationship between lymphocyte counts and PK.

One study has investigated peri-transplant alemtuzumab concentrations in relation to outcome of HSCT in 105 patients aged 0.3 to 27.2 years with non-malignant disease (29). The investigators identified that a very low concentration of alemtuzumab <0.15 μg/mL led to a lower incidence of mixed chimerism and better T-cell recovery but at the same time led to a higher incidence of acute GvHD. The authors did not investigate overall exposure of alemtuzumab as a predictor for outcome.

There are significant differences between ATG (Thymoglobulin^®^) and alemtuzumab in terms of PK and PD. Most striking is the difference in population clearance, which is 10- and 2-times lower for alemtuzumab than Thymoglobulin^®^ when measured by as linear clearance or as the maximum elimination rate in saturable clearance, respectively (22, 26). Patient-to-patient variability is also considerably higher for alemtuzumab than Thymoglobulin^®^ (26). Furthermore, the clearance of Thymoglobulin^®^ is higher in patients with higher lymphocyte counts, while this is not a predictor for alemtuzumab PK. In terms of PD, the so-called lympholytic level is significantly lower with alemtuzumab (0.1 μg/mL) than ATG (1.0 AU/mL) (30, 31). Due to lower clearance, the fraction of exposure to alemtuzumab occurring after infusion of the graft is higher after standard dosages and infusion timing. Therefore, alemtuzumab may be present at lympholytic levels for longer than is ATG, and its PK is less predictable.

## Summary

While there are still controversies regarding the role of serotherapy in transplantation for paediatric ALL and variations in practise around the world, studies continue to elucidate optimal timings and doses per agent. In addition, research into PK modelling and TDM continue to improve our knowledge regarding the correct dosing of each agent for the paediatric population, allowing clinicians to better tailor dose to specific patient characteristics. Pharmacogenetics are likely to play less of a role in serotherapy dosing than they do in the dosing of chemotherapy drugs used for conditioning. TDM for serotherapy has challenges and will likely be done only in specialised centres focused on research aimed at predicting population data and recommending dosing for paediatric patients.

## Author Contributions

All authors contributed to substantial sections of this manuscript, reviewed, and approved the completed document.

## Conflict of Interest

This study received funding from St. Anna Kinderkrebsforschung GmbH. The funder was not involved in the study design, collection, analysis, interpretation of data, the writing of this article or the decision to submit it for publication. The authors declare that the research was conducted in the absence of any commercial or financial relationships that could be construed as a potential conflict of interest.

## Publisher's Note

All claims expressed in this article are solely those of the authors and do not necessarily represent those of their affiliated organizations, or those of the publisher, the editors and the reviewers. Any product that may be evaluated in this article, or claim that may be made by its manufacturer, is not guaranteed or endorsed by the publisher.

## References

[1] Granot N Storb R. History of hematopoietic cell transplantation: challenges and progress. Haematologica. (2020) 105:2716–29. 10.3324/haematol.2019.245688 33054108 PMC7716373

[2] Storb R Floersheim GL Weiden PL Graham TC Kolb HJ Lerner KG. Effect of prior blood transfusions on marrow grafts: abrogation of sensitization by procarbazine and antithymocyte serum. J Immunol. (1974) 112:1508–16. 4592604

[3] Storb R Gluckman E Thomas ED Buckner CD Clift RA Fefer A. Treatment of established human graft-versus-host disease by antithymocyte globulin. Blood. (1974) 44:56–75. 10.1182/blood.V44.1.57.57 4275768

[4] Storb R Etzioni R Anasetti C Appelbaum FR Buckner CD Bensinger W. Cyclophosphamide combined with antithymocyte globulin in preparation for allogeneic marrow transplants in patients with aplastic anemia. Blood. (1994) 84:941–9. 10.1182/blood.V84.3.941.bloodjournal843941 8043876

[5] Popow I Leitner J Grabmeier-Pfistershammer K Majdic O Zlabinger GJ Kundi M. A comprehensive and quantitative analysis of the major specificities in rabbit antithymocyte globulin preparations. Am J Transplant. (2013) 13:3103–13. 10.1111/ajt.12514 24168235

[6] Mohty M. Mechanisms of action of antithymocyte globulin: T-cell depletion and beyond. Leukemia. (2007) 21:1387–94. 10.1038/sj.leu.2404683 17410187

[7] Green K Pearce K Sellar RS Jardine L Nicolson PLR Nagra S. Impact of alemtuzumab scheduling on graft-versus-host disease after unrelated donor fludarabine and melphalan allografts. Biol Blood Marrow Transplant. (2017) 23:805–12. 10.1016/j.bbmt.2017.02.007 28212937 PMC6588535

[8] Anasetti C Logan BR Lee SJ Waller EK Weisdorf DJ Wingard JR. Peripheral-blood stem cells versus bone marrow from unrelated donors. N Engl J Med. (2012) 367:1487–96. 10.1056/NEJMoa1203517 23075175 PMC3816375

[9] Pulsipher MA Langholz B Wall DA Schultz KR Bunin N Carroll WL. The addition of sirolimus to tacrolimus/methotrexate GVHD prophylaxis in children with ALL: a phase 3 Children's Oncology Group/Pediatric Blood and Marrow Transplant Consortium trial. Blood. (2014) 123:2017–25. 10.1182/blood-2013-10-534297 24497539 PMC3968388

[10] Wagner JE Jr Eapen M Carter S Wang Y Schultz KR Wall DA. One-unit versus two-unit cord-blood transplantation for hematologic cancers. N Engl J Med. (2014) 371:1685–94. 10.1056/NEJMoa1405584 25354103 PMC4257059

[11] Peters C Dalle JH Locatelli F Poetschger U Sedlacek P Buechner J. Total body irradiation or chemotherapy conditioning in childhood ALL: a multinational, randomized, noninferiority Phase III study. J Clin Oncol. (2021) 39:295–307. 10.1200/JCO.20.02529 33332189 PMC8078415

[12] Peters C Schrappe M von Stackelberg A Schrauder A Bader P Ebell W. Stem-cell transplantation in children with acute lymphoblastic leukemia: A prospective international multicenter trial comparing sibling donors with matched unrelated donors-The ALL-SCT-BFM-2003 trial. J Clin Oncol. (2015) 33:1265–74. 10.1200/JCO.2014.58.9747 25753432

[13] Locatelli F Bernardo ME Bertaina A Rognoni C Comoli P Rovelli A. Efficacy of two different doses of rabbit anti-T-lymphocyte globulin to prevent graft-versus-host disease in children with haematological malignancies transplanted from an unrelated donor: a multicentre, randomised, open-label, phase 3 trial. Lancet Oncol. (2017) 18:1126–36. 10.1016/S1470-2045(17)30417-5 28705454

[14] Kang HM Kim SK Lee JW Chung NG Cho B. Efficacy of low dose antithymocyte globulin on overall survival, relapse rate, and infectious complications following allogeneic peripheral blood stem cell transplantation for leukemia in children. Bone Marrow Transplant. (2021) 56:890–9. 10.1038/s41409-020-01121-9 33199818

[15] Bonifazi F Rubio MT Bacigalupo A Boelens JJ Finke J Greinix H. Rabbit ATG/ATLG in preventing graft-versus-host disease after allogeneic stem cell transplantation: consensus-based recommendations by an international expert panel. Bone Marrow Transplant. (2020) 55:1093–102. 10.1038/s41409-020-0792-x 31969678 PMC7269907

[16] Admiraal R van Kesteren C Jol-van der Zijde CM Lankester AC Bierings MB Egberts TC. Association between anti-thymocyte globulin exposure and CD4+ immune reconstitution in paediatric haemopoietic cell transplantation: a multicentre, retrospective pharmacodynamic cohort analysis. Lancet Haematol. (2015) 2:e194–203. 10.1016/S2352-3026(15)00045-9 26688094

[17] Michel G Galambrun C Sirvent A Pochon C Bruno B Jubert C. Single- vs double-unit cord blood transplantation for children and young adults with acute leukemia or myelodysplastic syndrome. Blood. (2016) 127:3450–7. 10.1182/blood-2016-01-694349 27099151

[18] Eapen M Kurtzberg J Zhang MJ Hattersely G Fei M Mendizabal A. Umbilical cord blood transplantation in children with acute leukemia: Impact of conditioning on transplantation outcomes. Biol Blood Marrow Transplant. (2017) 23:1714–21. 10.1016/j.bbmt.2017.06.023 28684372 PMC5605440

[19] Qin BZ Zhang C Zhang R Wang L. Role of antithymocyte globulin in patients with hematologic diseases undergoing umbilical cord blood transplantation: a systematic review and meta-analysis. Clin Transplant. (2020) 34:e13876. 10.1111/ctr.13876 32277839

[20] de Koning C Admiraal R Nierkens S Boelens JJ. Immune reconstitution and outcomes after conditioning with anti-thymocyte-globulin in unrelated cord blood transplantation; the good, the bad, and the ugly. Stem Cell Investig. (2017) 4:38. 10.21037/sci.2017.05.02 28607912 PMC5460145

[21] Jol-van der Zijde CM Bredius RG Jansen-Hoogendijk AM Raaijmakers S Egeler RM Lankester AC. IgG antibodies to ATG early after pediatric hematopoietic SCT increase the risk of acute GVHD. Bone Marrow Transplant. (2012) 47:360–8. 10.1038/bmt.2011.166 21892212

[22] Admiraal R van Kesteren C. Jol-van der Zijde CM van Tol MJ Bartelink IH Bredius RG. Population pharmacokinetic modeling of Thymoglobulin((R)) in children receiving allogeneic-hematopoietic cell transplantation: towards improved survival through individualized dosing. Clin Pharmacokinet. (2015) 54:435–46. 10.1007/s40262-014-0214-6 25466602

[23] Oostenbrink LVE. Jol-van der Zijde CM Kielsen K Jansen-Hoogendijk AM Ifversen M Muller KG. Differential elimination of anti-thymocyte globulin of Fresenius and genzyme impacts T-Cell reconstitution after hematopoietic stem cell transplantation. Front Immunol. (2019) 10:315. 10.3389/fimmu.2019.00315 30894854 PMC6414431

[24] Admiraal R Nierkens S Bredius R Bierings M van Vliet I Yurda ML. Prospective open-label phase II trial of individualized anti-thymocyte globulin for improved T-cell reconstitution after pediatric allogeneic hematopoietic cell transplantation: the parachute-study. Biol Blood Marrow Transplant. (2020) 26:S33–S4. 10.1016/j.bbmt.2019.12.577

[25] Sachs UJ Socher I Braeunlich CG Kroll H Bein G Santoso S. variable number of tandem repeats polymorphism influences the transcriptional activity of the neonatal Fc receptor alpha-chain promoter. Immunology. (2006) 119:83–9. 10.1111/j.1365-2567.2006.02408.x 16805790 PMC1782336

[26] Admiraal R Jol-van der Zijde CM Furtado Silva JM Knibbe CAJ Lankester AC Boelens JJ. Population pharmacokinetics of alemtuzumab (Campath) in pediatric hematopoietic cell transplantation: towards individualized dosing to improve outcome. Clin Pharmacokinet. (2019) 58:1609–20. 10.1007/s40262-019-00782-0 31131436 PMC6885503

[27] Fukuda T Emoto C Marsh RA Neumeier L Vinks AA Mehta PA. Precision dosing of alemtuzumab: Population pharmacokinetic modeling in pediatric patients undergoing allogeneic hematopoietic cell transplantation for non-malignant diseases. Blood. (2016) 128:2203. 10.1182/blood.V128.22.2203.220316462024

[28] Dong M Emoto C Fukuda T Arnold DE Mehta PA Marsh RA. Model-informed precision dosing for alemtuzumab in paediatric and young adult patients undergoing allogeneic haematopoietic cell transplantation. Br J Clin Pharmacol. (2021). 10.1111/bcp.14955 34182590

[29] Marsh RA Lane A Mehta PA Neumeier L Jodele S Davies SM. Alemtuzumab levels impact acute GVHD, mixed chimerism, and lymphocyte recovery following alemtuzumab, fludarabine, and melphalan RIC HCT. Blood. (2016) 127:503–12. 10.1182/blood-2015-07-659672 26644451

[30] Waller EK Langston AA Lonial S Cherry J Somani J Allen AJ. Pharmacokinetics and pharmacodynamics of anti-thymocyte globulin in recipients of partially HLA-matched blood hematopoietic progenitor cell transplantation. Biol Blood Marrow Transplant. (2003) 9:460–71. 10.1016/S1083-8791(03)00127-7 12869960

[31] Morris EC Rebello P Thomson KJ Peggs KS Kyriakou C Goldstone AH. Pharmacokinetics of alemtuzumab used for *in vivo* and *in vitro* T-cell depletion in allogeneic transplantations: relevance for early adoptive immunotherapy and infectious complications. Blood. (2003) 102:404–6. 10.1182/blood-2002-09-2687 12623851

